# The mechanisms of calcium-catalyzed graphenization of cellulose and lignin biochars uncovered

**DOI:** 10.1038/s41598-023-38433-x

**Published:** 2023-07-14

**Authors:** Théotime Béguerie, Elsa Weiss-Hortala, Nathalie Lyczko, Ange Nzihou

**Affiliations:** 1 IMT Mines Albi, CNRS, Centre RAPSODEE, Université de Toulouse, Campus Jarlard, Route de Teillet, .81013 Albi Cedex 09, France; 2 Institute of Multidisciplinary Research for Advanced Materials (IMRAM), Tohoku University, Sendai, Japan; 3 School of Engineering and Applied Science, Princeton University, Princeton, NJ 08544 USA; 4 Andlinger Center for Energy and the Environment, Princeton University, Princeton, NJ 08544 USA

**Keywords:** Synthesis of graphene, Energy storage, Ecology, Graphene

## Abstract

A recent study has shown that highly crystalline graphene-based materials can be obtained from poorly organized carbon precursors using calcium as a non-conventional catalyst. XRD and TEM analyses of calcium-impregnated cellulose and lignin biochars showed the formation of well-ordered graphenic structures (L_c_ > 7 nm, d_002_ < 0.345 nm) above 1200 °C, far below the standard graphenization temperatures (T > 2000 °C). Herein, we propose new insights on the mechanism controlling the formation of highly graphenic biochars using Ca as a catalyst. We postulate that the calcium-catalyzed graphenization occurs through the formation of a metastable calcium carbide by reaction between CaO particles and amorphous carbon between 1000 and 1200 °C. CaC_2_ decomposes into calcium vapor and a graphenic shell covering the CaC_2_ particles as confirmed by TEM analysis. The thickness and planarity of the graphenic shell increase with the CaC_2_ initial particle size (between 20 and 200 nm), and its growth is controlled by the diffusion of the calcium vapor through the graphene layer. A much effective graphenization was obtained for the lignin biochars compared to cellulose, with L_c_ > 10 nm and d_002_ < 0.340 nm, attributed to the insertion of sulfur in the graphenic shells, which favors their ruptures and the decomposition of CaC_2_ into graphene. We believe that these findings would enable the reduction of costs and environmental impact of graphene-based materials synthesis using cheap and abundant renewable feedstocks and catalysts as well.

## Introduction

Graphene is a bidimensional carbon material composed of sp^2^ hybridized carbon atoms arranged in a hexagonal lattice. It is characterized by high electrical conductivity (~ 10^6^ s m^−1^), mechanical resistance (tensile strength ≈ 130 GPa) and specific surface area (~ 2675 m^2^ g^−1^)^1,2^. Therefore, Graphene and graphene-based materials such as fullerene, carbon nanotube or graphite are considered for applications in various advanced fields such as supercapacitors^3–5^, electronics^6^, energy storage^7^ and medical devices^8,9^. Graphene is currently synthetized by either top-down or bottom-up processes that usually require complex mechanical, chemical or thermal treatments^10–12^. In addition, most of the standard carbon precursors for graphene synthesis involves non-sustainable fossil or petroleum-based resources, which deepens the environmental cost of graphene and graphene-based materials synthesis.

Over the last years, the use of lignocellulosic biomass as a precursor for graphene synthesis has attracted lot of attention due to its abundance, renewability, and cheap cost. However, lignocellulosic bioresources, which are a complex mix of cellulose, hemicelluloses and lignin with inorganic elements, are “non-graphitizing” carbons, which means that they produced short and irregularly stacked graphene sheets (turbostratic carbon) even after carbonization at high temperatures (T > 2000 °C)^13–16^. Therefore, graphenization corresponds to the formation and growth of graphene layers from disordered carbon to 2D carbon materials with a slight 3D character (turbostratic), while graphitization refers to a regular crystalline 3D structure^17^. Nonetheless, previous studies have reported that the doping of the lignocellulosic biomass precursor with carefully-selected species improves graphenization of non-graphitizing carbons to highly crystalline graphene-based materials at relatively low temperatures (T < 1000 °C). Hoekstra et al*.* and Sevilla et al*.* both obtained highly crystalline graphenic carbons from cellulose at 800 and 900 °C using nickel^18,19^, while Yan et al*.,* Gong et al*.* and Thompson et al*.* used iron to produce multilayer graphenic materials below 1000 °C from kraft lignin, bamboo and softwood sawdust respectively^20–22^. Most of the articles about catalytic graphenization focused on transition metals, especially iron, cobalt and nickel for their graphenization efficiency and abundance^19–27^. Nonetheless, old research conducted in the 80’s, summarized by Oya et al.^28^, have revealed that multiple elements, in particular the alkaline earth metals such as calcium, can be efficient catalysts for the graphenization of various carbon ressources^29,30^. This latter route was no longer considered until a recent study from our team. We have confirmed the catalytic activity of calcium on the graphenization of cellulose biochar carbonized at 1800 °C^31^. In summary, impregnation of the bioresource with calcium led to the formation of a highly graphenic domain in the biochar, whose proportion rises with the calcium loading. As opposed to the standard transition metals catalysts (Fe, Ni, Co), calcium is more environmental-friendly, abundant and cheaper, and could therefore attract more attention in future works in the field of graphenic carbon materials.

The fundamental understanding of the mechanism of catalytic graphenization is a key issue to further optimize the process of green synthesis of graphene from bioresources and to obtain graphenic materials with tunable properties. Several mechanisms have been proposed for transition metals, and the most widely accepted ones are the *carbide formation–decomposition* and the *dissolution–precipitation* mechanisms. In the *carbide formation–decomposition* mechanism, the catalyst reacts with the amorphous carbon to form a metastable carbide intermediate that decomposes into graphene at high temperature^28,32^. In the *dissolution–precipitation* mechanism, the amorphous carbon dissolves in the molten metal that becomes supersaturated before precipitating as defect-free graphene during cooling^18^. Gomez-Martin et al*.* studied the iron-catalyzed graphenization of non-graphitizing carbon with in situ X-ray diffraction and ex situ total scattering experiments, and found the formation and decomposition into graphene of an iron carbide upon cooling at temperature as low as 800 °C^24^. More recently, Ghogia et al*.* investigated the catalyzed graphenization of iron-impregnated cellulose biochars and found that both the particles size and degree of reduction of the iron catalyst favored by thermal heating promote the biochar graphenization^27^.

To the best of our knowledge, the mechanism controlling the catalytic graphenization of cellulose and lignin using calcium has not been discussed in the literature. Hirano et al*.* reported in the 70s the formation of an unstable calcium carbide and its decomposition into crystalline graphenic structures above 1300 °C during the carbonization of polyvinylchloride coke with calcium oxide^29^. More recently, Yang et al*.* used melamine–formaldehyde resin as carbon precursor and CaCO_3_ nanoparticles as template to produce graphite-like nano-shells below 1300 °C, suggesting that graphenic structures develop around the catalyst particles^33^. Hence, the comprehensive mechanism driving the calcium-catalyzed graphenization lacks understanding.

**Figure 1:**
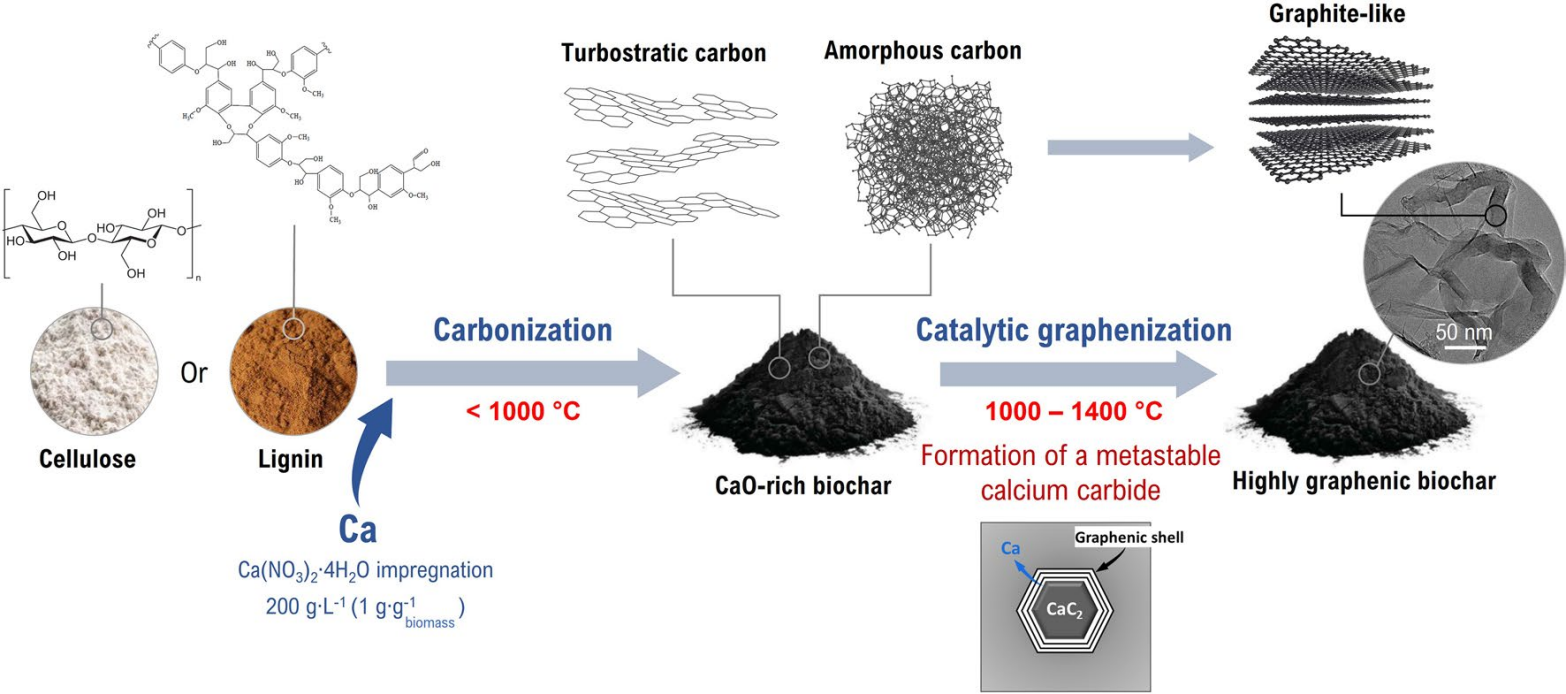
Schematic representation of the synthesis of highly graphenic biochar using calcium.

In this context, this work aims at providing new insights on the comprehensive mechanism of calcium-catalyzed graphenization. To this end, we have carbonized calcium-impregnated commercial cellulose and lignin, two of the main polymers of a lignocellulosic bioresource, between 1000 and 1800 °C (Fig. 1). The carbon organization of the resulting biochars was characterized using X-ray diffraction (XRD) and transmission electron microscopy (TEM). From the results, we have highlighted the starting temperature of the catalytic activity of calcium, and we propose an approach describing the fundamental graphenization mechanism as a first contribution to the state of the art in the field.

## Results

### Temperature-dependance of the biochars graphenization

**Figure 2:**
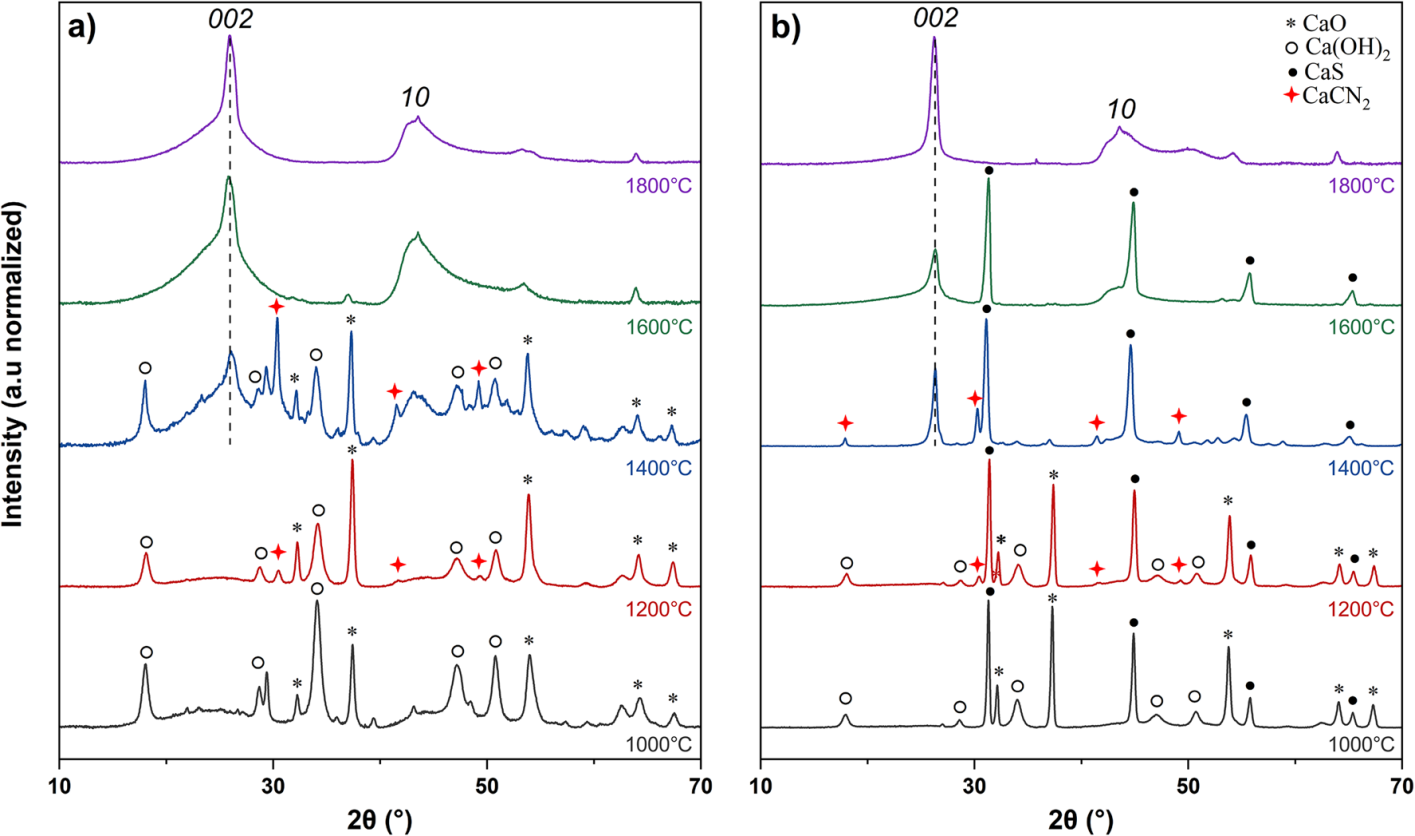
XRD patterns (λ = 1.542 Å) of biochars carbonized at different temperatures: (**a**) calcium-impregnated cellulose biochars, (**b**) calcium-impregnated lignin biochars.

The XRD spectra of the calcium-impregnated cellulose biochars at different carbonization temperatures are presented in Fig. 2a. For the biochars prepared at 1000 and 1200 °C, multiple diffraction peaks of calcium species are observed, which are attributed to CaO (* symbols) and Ca(OH)_2_ (○ symbols) particles. As Ca(OH)_2_ decomposes into CaO and water around 500 °C^34^, the observed Ca(OH)_2_ likely results from the reaction of CaO particles with the room moisture after cooling. Application of the Scherrer equation indicates a slight decrease of the average particle diameter L_CaO_, going from L_CaO_ = 21.70 nm at 1000 °C to 21.21 nm at 1400 °C. No distinctive carbon *002* peak is visible at these carbonization temperatures, which indicates that the carbon matter in these biochars is either amorphous or highly disorganized in this temperature range (1000–1200 °C).

However, a sharp diffraction peak appears at the carbon *002* position (2θ ≈ 26°) and confirmed the formation of a graphenic domain from 1400 °C. From these observations, the graphenization starts between 1200 and 1400 °C, which is in agreement with previous observations from Hirano et al*.*^29^, who reported the formation of a graphenic domain in CaO-doped polyvinylchloride coke above 1300 °C. The biochar carbonized at 1400 °C still contains multiple calcium species, and the diffraction peaks of calcium cyanamide (CaCN_2_) are visible (red  symbols). Calcium cyanamide is also observed at 1200 °C, and should result from reactions of calcium species with the atmospheric nitrogen.

At 1600 and 1800 °C, the diffraction peaks of the calcium species disappear. This could be attributed to the volatilization of the calcium species or their entrainment in the flue-gas stream^35^. The carbon *002* peak is sharp but asymmetric, which indicates the coexistence of two carbon phases, one highly turbostratic as observed for non-graphitizing carbons, and another highly crystalline. The *002* peak becomes thinner with the temperature, because of the improvement of the crystalline organization in the turbostratic domain, or because of stackings and junctions of the highly graphenic structures.

A similar trend is observed for the lignin biochars, with the apparition of a graphenic domain above 1400 °C (Fig. 2b). Calcium cyanamide is also detected at 1200 and 1400 °C, and most of the CaO and Ca(OH)_2_ particles have disappeared at 1400 °C unlike the cellulose biochar. The main difference with the cellulose biochars is the apparition of the diffraction peaks of calcium sulfide (CaS). Kraft lignin used in this study is indeed rich in sulfur (14.2 g kg^−1^) that comes from the industrial extraction process. It has previously been reported that calcium oxide can interact with sulfuric acids vapors formed during pyrolysis to produce calcium sulfate^36^, which is then reduced to calcium sulfide around 900 °C^37,38^.

Even though CaS is formed as soon as 900 °C, below the apparent temperature of formation of the graphenic structures, sulfur does not seem to have inhibit or reduce the catalytic activity of calcium. The sizes of the CaS and CaO particles in the lignin biochars significantly decrease with temperature, from L_CaS_ = 34.53 nm to L_CaS_ = 18.97 nm between 1000 and 1600 °C, and from L_CaO_ = 31.50 nm to L_CaO_ = 27.34 nm between 1000 and 1200 °C, but remained higher than for the CaO particles in the cellulose biochars in the same temperature ranges (L_CaO_ ≈ 22 nm). This trend is opposed to the standard graphenization catalysts like iron, where sintering of the metal particles with temperature was reported^39^.

The crystallite sizes L_c_ and d_002_ of the highly crystalline graphenic domains in the biochars measured from the XRD spectra are summarized in Table 1. Higher crystallite size L_c_ and lower interlayer spacing d_002_ are obtained at 1400 °C. The high-intensities peaks of the calcium species in the XRD spectra may have hidden the contribution of the amorphous carbon to the apparent *002* peak, resulting in a thinner *002* peak. L_c_ increases and d_002_ decreases between 1600 and 1800 °C, which is consistent with the literature that indicates an improvement of the graphenization with the temperature^39^. The graphenic domain in the lignin biochars is more developed than in the cellulose biochar, with a greater L_c_ and lower d_002_. This enhancement could be explained by the slightly greater calcium loading in the initial lignin (4.72 wt.% vs 4.39 wt.%), from a positive effect of sulfur on the graphenization, or from the larger size of the calcium particles, as the metal particles size is known to improve the crystallite sizes of carbon^39^. To further investigate the effect of calcium on the carbon organization, the structure of the biochars was studied using TEM.

**Table 1:** Crystallite sizes L_c_ and d_002_ of the graphenic domains in the biochars between 1400 and 1800 °C.

	Cellulose			Lignin		
	1400 °C	1600 °C	1800 °C	1400 °C	1600 °C	1800 °C
L_c_ (nm)	7.694	6.79	7.88	17.41	10.29	12.17
d_002_ (nm)	0.341	0.344	0.343	0.339	0.339	0.340

### Observation of the carbon structure

**Figure 3:**
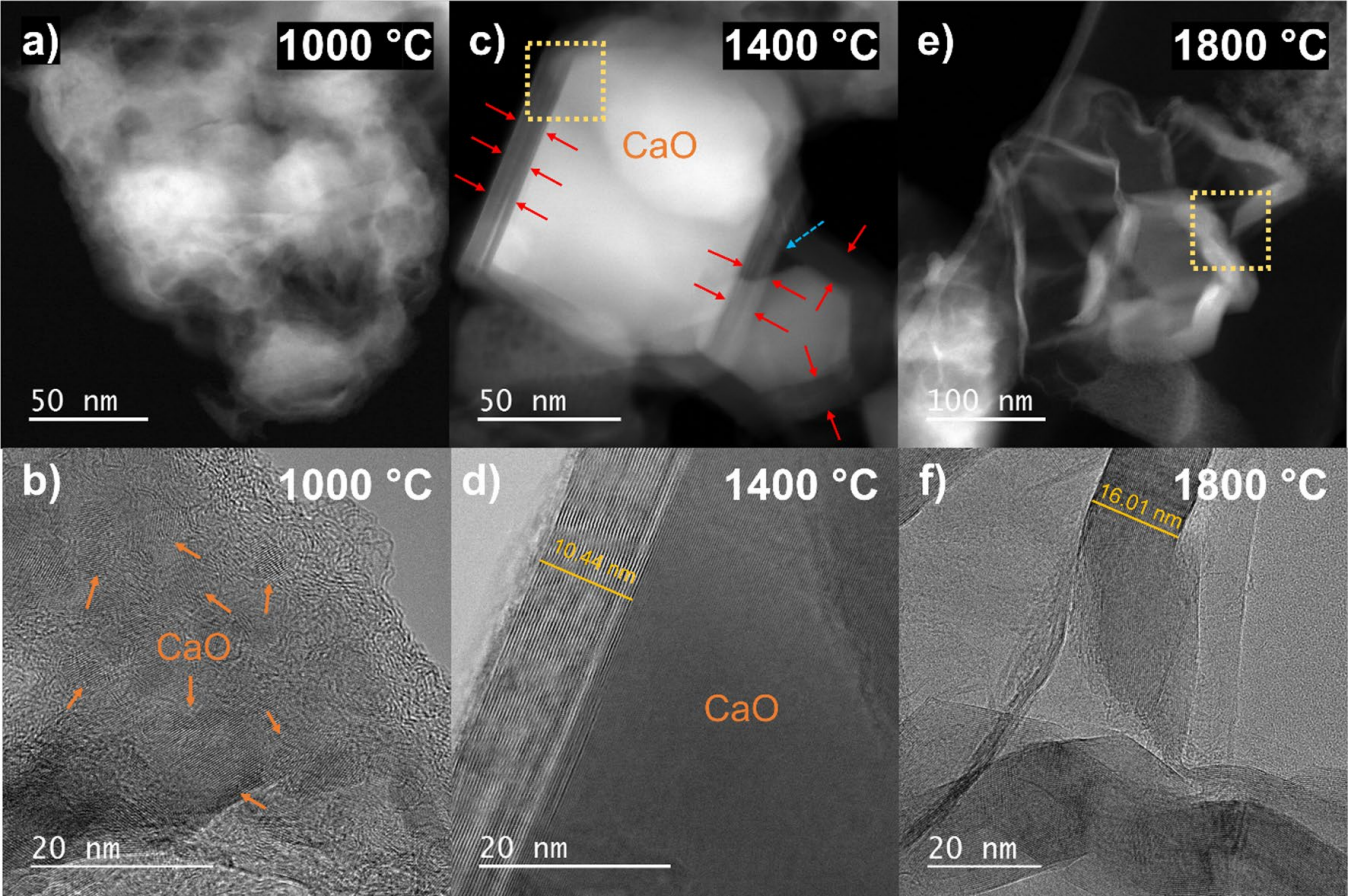
TEM images of the calcium-impregnated cellulose biochars at different temperatures: (**a**) 1000 °C dark field, (**b**) 1000 °C HRTEM, (**c**) 1400 °C dark field, (**d**) 1400 °C HRTEM, (**e**) 1800 °C dark field, (**f**) 1800 °C HRTEM.

TEM images of the calcium-impregnated cellulose biochars at different temperatures are presented in Fig. 3. The corresponding EDX analyses and additional images are presented in the Supplementary materials. Figure 3a, b, acquired at 1000 °C, show a poorly organized carbon matrix, typical of a non-graphitizing carbon, which confirms the XRD results that showed no catalytic effect of calcium at this temperature. Moreover, the TEM images reveal the presence of large amounts of CaO particles embedded in the carbon matrix.

However, the formation of a graphenic shell around a large calcium oxide particle is observed at 1400 °C (Fig. 3c, d). This graphenic shell is formed by the regular stacking of long-range graphene sheets and explains the high crystallite size L_c_ calculated from XRD. Even though the graphenic shell follows the edges of the particle, it is opened as it could be seen in the upper part of the image, which suggests that the CaO particle can partially slide outside of its shell, creating hollow spaces between the particle and the graphene sheets (blue arrow in Fig. 3c). Formation of a graphenic shell around a catalyst particle has already been observed with iron^23,39^. For this catalyst, the dimensions and orientations of the graphenic structures are directly linked to the geometry of the metal particle. Therefore, a large particle with planar facets, as observed in Fig. 3c, will form well-ordered and developed graphenic structures, while a small particle with an irregular shape will result in short and poorly ordered graphenic structures^39^ (see Fig. S3). The CaO particles are formed in situ during the carbonization (liquid impregnation), and therefore their sizes are not controlled, which results in heterogenous graphenic structures.

At 1800 °C, calcium particles are no longer observed due to their volatilization or entrainment in the flue-gas stream, and the carbon matrix has a crumpled-paper morphology composed of several cavity-shape graphenic structures (Fig. 3e, f). These structures have a diameter between 20 and 200 nm, in accordance with the dimensions of the graphenic shells observed at 1400 °C. Therefore, we can assume that these structures correspond to the graphenic shell leftovers after removal of the CaO core. Without a solid particle as a core, the graphenic shells can easily deform, which causes a broadening of the diffraction peaks and explain the higher crystallite size L_c_ at 1400 °C.

**Figure 4:**
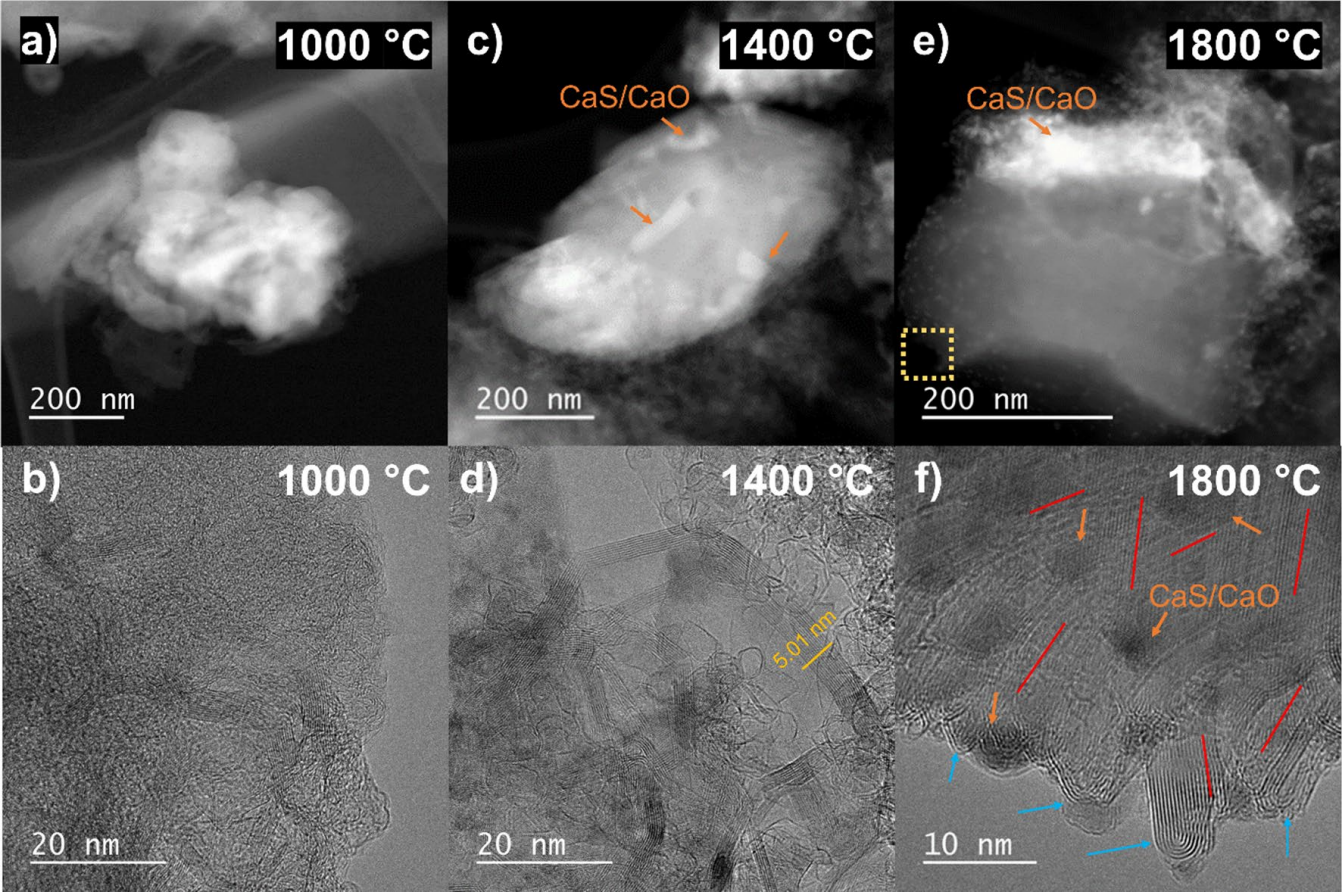
TEM images of the calcium-impregnated lignin biochars at different temperatures: (**a**) 1000 °C dark field, (**b**) 1000 °C HRTEM, (**c**) 1400 °C dark field, (**d**) 1400 °C HRTEM, (**e**) 1800 °C dark field, (**f**) 1800 °C HRTEM.

Figure 4 presents the TEM results for the calcium-impregnated lignin biochars. At 1000 °C, the carbon matrix is highly disordered with many calcium particles (CaO and CaS) embedded in (Fig. 4a, b). Some additional mineral elements in smaller proportions were detected, namely potassium, silica and iron. In opposition to the cellulose biochar, some crystalline graphenic structures are observed in addition to the disordered carbon. This can be attributed to an early start of the calcium-catalyzed graphenization for the lignin biochar triggers by sulfur, or to a catalytic effect of the inorganic species inherent to the lignin bioresource (Table S1). Indeed, the commercial lignin used in this study contains some iron (0.4 g kg^−1^), which is known to catalyze graphenization at temperature as low as 800 °C^19^. However, the biochar is mostly disorganized overall, as no crystalline graphenic structures *002* peak is observed in the XRD spectrum.

The TEM images of the lignin biochars carbonized at 1400 and 1800 °C both show peculiar carbon structures riddle with CaO and CaS nanoparticles (Fig. 4c, e, f, see Figs. S4 and S5 for the EDX analyses). They all present irregular and unique shapes and are few of hundred nanometers large. A zoom on one of these structures (Fig. 4f) show that they are composed of tens of stacked graphene sheets with embedded CaO and CaS nanoparticles. The graphene sheets present various orientations (highlighted by red lines) but remain mostly flat for tens of nanometers in width, despite some slight curvatures and U-shaped folds at the edges of the graphenic structure (blue arrows). The better stacking of the graphene sheets in these structures, unique to the lignin biochars, is in agreement with the greater crystallite size L_c_ and lower interlayer spacing d_002_ shown with the XRD analysis. The presence of CaS particles on the graphenic structures confirm that sulfur did not inhibit the catalytic activity of calcium and may have intervene in the graphenization mechanism. However, some graphenic structures without CaS particles (Fig. 4d), as well as lone CaO, particles were observed in the lignin biochars. This suggests that some calcium particles have catalyzed the lignin biochars graphenization without interaction with sulfur, in the same way than with the cellulose biochars. It is relevant to note that no sodium species were detected in the lignin biochars despite its high content (51.5 g kg^−1^) in the commercial lignin employed. Na is known to volatilize below 1000 °C during biomass pyrolysis^40^. It is likely that Na was already vaporized from the lignin biochars before the start of the graphenization, and therefore did not play any role in the Ca-catalyzed graphenization process.

## Discussion

Highly graphenic structures can be formed by impregnation of cellulose and lignin with calcium at much lower temperatures than with the standard processes. As these main polymers of lignocellulosic biomass and calcium are both cheap and abundant resources, this novel approach would drastically reduce the cost and environmental impact of graphene-based materials production. That is why a precise understanding of the graphenization mechanism is required to control and improve the desired properties of the formed carbon materials. However, apart for scarce studies in the literature^29,30,33^, very limited information is known about the calcium-catalyzed graphenization mechanism. Based on our findings, we can propose some new insights on how calcium help to rearrange the carbon atoms into graphenic structures. As the cellulose and lignin biochars presented noticeably different graphenic structures, these two bioresources will be discuss separately, starting from cellulose.

From the XRD and TEM analyses, temperature appears to be the main factor driving the catalytic graphenization. Below 1200 °C, no catalytic effect of calcium is observed, and the calcium is mainly under the form of CaO particles embedded in an amorphous carbon matrix. However, formation of graphenic structures is observed around calcium oxide particles at 1400 °C on the TEM images. This observation is similar to the literature on iron-catalyzed graphenization. It is explained that the amorphous carbon dissolves in the metal catalyst to form an intermediary metastable carbide, and then precipitates into a graphenic shell around the catalyst upon cooling^24,27^. This mechanism fits well with calcium, as calcium oxide is known to react with carbon to form a calcium carbide around 1300 °C^41^:

1 $$CaO+{3C}_{amorphous}\to Ca{C}_{2}+CO$$

CaC_2_ is known to be highly unstable^42^, and therefore it decomposes quickly at higher temperatures. Previous reports on the desulfurization of metal by CaC_2_ and on its thermal behavior suggests that CaC_2_ decomposes above 1200 °C to produce calcium vapor and a graphene layer around the CaC_2_ particle (reaction 2)^42–44^. The formation of calcium vapor may explain the disappearance of calcium in the biochars above 1200 °C. According to literature, this reaction is mainly driven by the diffusion of the Ca vapor through the graphenic shell^43^, and the reaction stops when the CaC_2_ particle is fully enveloped with a thick graphene layer.

2 $$Ca{C}_{2}\to Ca+{2C}_{graphene}$$

However, neither CaC_2_ or metallic Ca were observed during the XRD and TEM analyses, which can be explained by their reactions with moisture and air when the samples have leaved the furnace to produce calcium oxide or calcium hydroxide^33^.

Nonetheless, the observation of calcium cyanamide can served as an undirect proof of CaC_2_ formation, as CaC_2_ was reported to react with atmospheric nitrogen above 1000 °C to form calcium cyanamide and a graphene phase according to the reaction^30,33,45^:

3 $$Ca{C}_{2}+{N}_{2}\to CaC{N}_{2}+{C}_{graphene}$$

This suggests the formation of CaC_2_ in the biochars between 1000 and 1200 °C, slightly below the standard formation temperature reported in literature (T ≥ 1300 °C)^41^. As such, calcium catalyzes the biochars graphenization by formation of a metastable CaC_2_, followed by its decomposition either into calcium vapor and graphene or calcium cyanamide and graphene in case of a nitrogen atmosphere. As no graphenic carbon was observed at 1200 °C on the XRD spectra of the cellulose and lignin biochars despite apparent formation of calcium cyanamide, it is postulated that the graphenization mainly occurs by the first mechanism. Working with a different inert atmosphere, such as argon, can help to conclude on the preferential mechanism.

Based on previous reports using more conventional catalysts, the metal carbide can either decompose during the cooling phase (“dissolution–precipitation” mechanism) as for Fe_3_C^24^, or during the heating phase (*formation–decomposition* mechanism) as for SiC^46^. As the experiments were carried out ex situ, it is difficult to conclude between the two mechanisms, but the formation of graphenic structures between 1200 and 1800 °C despite the volatilization of the calcium particles in this temperature range and the high instability of CaC_2_ tends to indicate that the catalytic graphenization happens during the heating phase.

**Figure 5:**
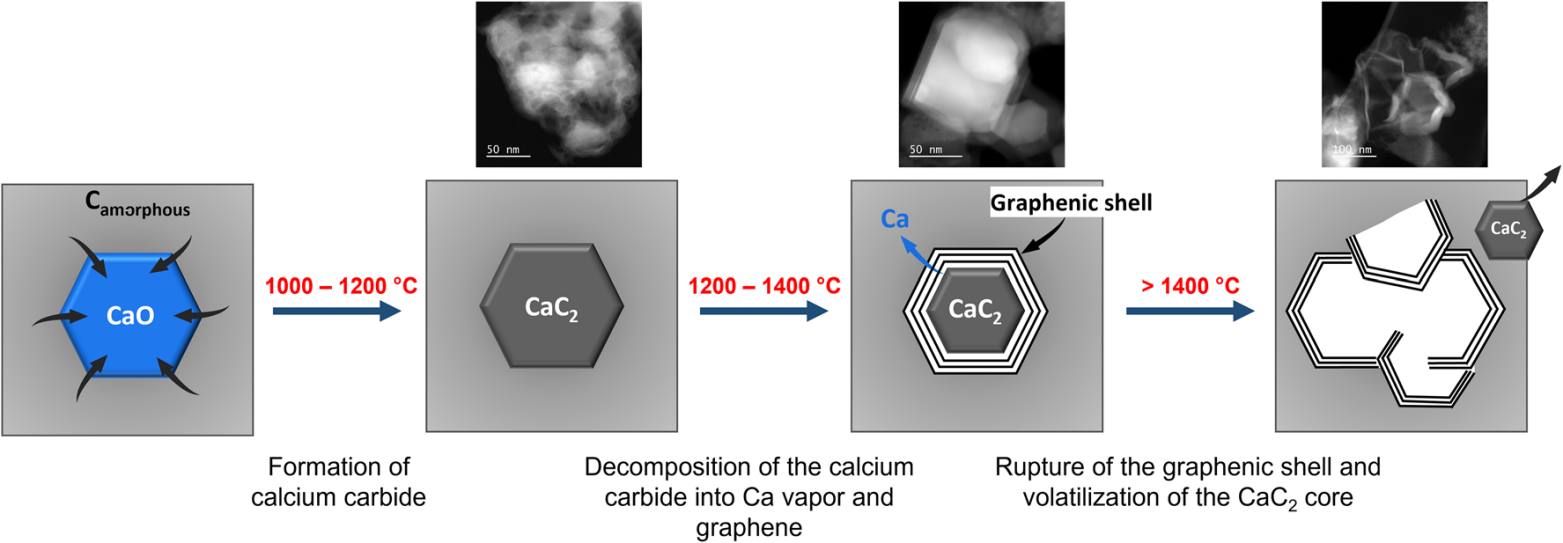
Schematic representation of the calcium-catalyzed graphenization.

Above 1400 °C, the unreacted CaC_2_ and CaCN_2_ particles may slide out from their graphenic shells and are entrained out of the biochars in the flue-gas stream. The graphenic shells agglomerate, which confers to the carbon matrix a crumpled-paper morphology. It is possible that some stackings and junctions of the graphenic structures happen, but their high curvatures and random orientations limit this possibility. Based on our results and on the literature, a schematic description of the calcium-catalyzed mechanism is proposed on Fig. 5.

In the case of the lignin biochars, the larger size of the CaO particles indicated by XRD (L_CaO_ = 31.5 nm vs 22 nm for cellulose at 1000 °C) may have led to the formation of bulkier CaC_2_ particles and larger graphenic structures as a result. The fine CaS nanoparticles observed in the TEM images may come from the reaction of the calcium vapor formed by reaction 2 with the sulfur linked to the carbon matrix (reaction 4)^43,44^:

4 $$Ca+S\to CaS$$

As explained before, reaction 2 is driven by the diffusion of calcium through the graphene layer around the CaC_2_ particle^43^, and its total encapsulation drastically limits the growth of the graphenic shell. It is possible that the graphenic shell may much easily break in the lignin biochars, possibly because of insertion of some sulfur in the graphene sheets, so that the CaC_2_ particle is always partially uncovered and ready to decompose. This could explain the shrinkage of the CaO and CaS particles with the temperature observed during the XRD analyses. Below 1200 °C, CaS is mainly formed from the reaction of initial large CaO particles with sulfuric acids vapors, but is produced at higher temperatures by reaction 4 from the calcium vapor produced by the CaC_2_ decomposition (reaction 2), which explains their fine dispersion near the graphenic structures. The CaC_2_ particles shrink as the graphenic structures develop and turn into small CaO particles after reaction with moisture when exiting the furnace. Additional research have to be carried out to better understand the role of sulfur in the mechanism of calcium-catalyzed graphenization.

## Conclusion

The comprehensive mechanism of the catalytic graphenization of non-graphitizing carbons using calcium was investigated by carbonizing calcium-impregnated cellulose and lignin between 1000 and 1800 °C. The formation of highly crystalline graphenic structures (L_c_ > 7 nm, d_002_ < 0.345 nm) was observed for both bioresources when carbonized at 1400 °C. TEM analysis showed a graphenic shell around the catalyst particles. Above 1400 °C, the calcium particles volatilize and the graphenic shells agglomerate, which confers a crumpled-paper morphology to the carbon matrix. More ordered graphenic structures were found in the lignin biochars (L_c_ > 10 nm, d_002_ < 0.340 nm), suggesting a greater effect of calcium on this bioresource.

To explain the catalytic formation of the graphenic structures, new insights have been brought in this study. We have postulated that CaO reacts with amorphous carbon to produce a metastable calcium carbide around 1200 °C, which decomposes at higher temperatures into calcium vapor and a graphenic shell around the CaC_2_ particle (20 to 200 nm in width). The growth of the graphenic shell stops when the CaC_2_ particle is fully enveloped with a thick graphene layer and when the calcium vapor can no longer diffuse through the graphene layer. The sulfur in the lignin biochars has favored the rupture of the graphenic shells, which promotes the decomposition of CaC_2_ into graphene.

We believe that these new insights to the body of knowledge on the mechanism of calcium-catalyzed graphenization may contribute to widen research opportunities in the field of green synthesis of innovative graphenic material using cheap, abundant and renewable resources.

## Methods

### Impregnation protocol and determination of the inorganic composition

Microcrystalline cellulose (CAS: 9004-34-6) and kraft lignin (CAS: 8068-05-1) provided by *Sigma Aldrich* were used as carbon precursors and were loaded with calcium by liquid impregnation. 40 g of cellulose and lignin were immersed in 200 ml of a Ca(NO_3_)_2_ 4H_2_O aqueous solution (respectively at 0.847 M and 0.381 M) and stirred for 6 h before filtration and drying at 105 °C for 24 h.

The inorganic compositions of the impregnated and non-impregnated bioresources were determined by inductively coupled plasma-optical emission spectroscopy (ICP-OES, Horiba Ultima 2). The calcium concentration in the impregnated bioresources was respectively 4.39 wt.% and 4.72 wt.% for cellulose and lignin. The detailed inorganic compositions of the bioresources are available in supplementary Table S1.

### Biochars preparation

The impregnated bioresources were first carbonized for 1 h at 800 °C under N_2_ with a heating ramp of 2 °C min^−1^ in a vertical tubular oven (carbolite vertical furnace) before uncontrolled cooling at room temperature. The resulting biochars were then carbonized for 1 h at a final temperature ranging from 1000 to 1800 °C (step of 200 °C) under N_2_ at 2 °C min^−1^ in a horizontal tubular furnace (Nabertherm RHTH 80/300/18).

### X-ray diffraction

The crystalline organization of the biochars was studied by X-ray diffraction using a PANalytical X’pert Pro MPD with a Cu-Kα radiation source (λ = 1.542 Å) and operating at 45 kV and 40 mA. Diffraction peaks were recorded at 0.5 ° s^−1^ in the range 10°–70° in 2θ.

The carbon *002* diffraction peak (2θ ≈ 24°) was used for the evaluation of the out-of-plane organization in the graphenic domain. The *002* peak was fitted with one or two pseudo-Voigt functions depending on its asymmetry to account for poorly organized carbon. The most intense diffraction peaks of the inorganic species were also fitted with a pseudo-Voigt and used for the determination of the inorganic particles sizes. The average stacking height L_c_ of the graphenic domain and inorganic particles sizes L_CaX_ (where CaX refers to a calcium specie) were all estimated using the Scherrer equation and the interlayer spacing d_002_ (average distance between two graphene layers in the graphenic domain) by Bragg’s law:

$$L \left(nm\right)=\frac{K\cdot \lambda }{\sqrt{{\beta }^{2}-{s}^{2}}\cdot cos\theta }$$

$${d}_{002}(nm)=\frac{\lambda }{2\cdot sin{\theta }_{002}}$$

where *λ* is the radiation wavelength (0.1542 nm), *θ* is the peak position, *K* is a constant taken as 0.89 for both L_c_ and L_CaX_, *β* is the Full Width at Half Maximum (FWHM, in radians) of the studied diffraction peaks and *s* the FWHM of a standard specimen (silica) to adjust for instrumental broadening.

### Transmission electron microscopy

The carbon structures were observed using High-Resolution Transmission Electron Microscopy (HRTEM, JEOL cold-FEG JEM-ARM200F) operated at 200 kV equipped with a probe Cs corrector reaching a spatial resolution of 0.078 nm. EDX spectra were recorded on a JEOL CENTURIO SDD detector. Biochars particles with a size below 50 μm were first dispersed in ethanol and then deposited on a copper grid with a carbon Lacey film.

## Supplementary Information

Supplementary Information.

## Data Availability

The datasets generated during and/or analyzed during the current study are available from the corresponding author on reasonable request.
