# Supplementary material for: The mechanisms of calcium-catalyzed graphenization of cellulose and lignin biochars uncovered
Source: Sci Rep. 2023 Jul 14;13:11390. doi: 10.1038/s41598-023-38433-x (PMC10349037; doi:10.1038/s41598-023-38433-x)
Supplement: Supplementary file 1 — Supplementary Information. [file 41598_2023_38433_MOESM1_ESM.pdf]

Supplementary information

The mechanisms of calcium-catalyzed graphenization of cellulose and lignin biochars uncovered

Théotime Béguerie, Elsa Weiss-Hortala, Nathalie Lyczko, Ange Nzihou

Inorganic composition ($mg{\cdot}kg^{-1}$)									
	**Al**	**Ca**	**Fe**	**K**	**Mg**	**Mn**	**Na**	**S**	**Si**
Non-impregnated cellulose	16	32	24	50	/	/	29	/	172
Ca-impregnated cellulose	221	43952	97	352	52	47	264	/	81
Non-impregnated lignin	213	497	144	3268	109	77	18768	15750	1313
Ca-impregnated lignin	264	47233	411	5470	325	321	51453	14203	1688

Table S1. Inorganic compositions of the non-impregnated and calcium-impregnated resources.

Formation of calcium sulfide from calcium oxide:

$$CaO + H_2SO_4 \rightarrow CaSO_4 + H_2O \quad (S1)$$

$$CaSO_4 + 2C_{char} \rightarrow CaS + 2CO_2 \quad (S2)$$

Reaction of calcium carbide with moisture:

$$CaC_2 + H_2O \rightarrow Ca(OH)_2 + C_2H_2 \quad (S3)$$

$$2Ca + O_2 \rightarrow 2CaO \quad (S4)$$

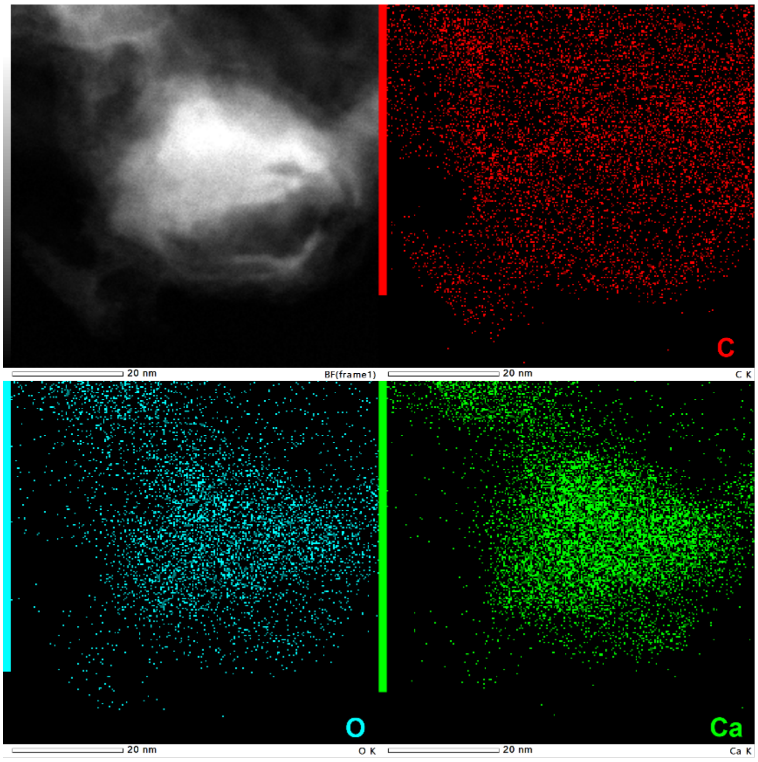

Figure S1. EDX analysis of the calcium-impregnated cellulose biochar carbonized at 1000 °C.

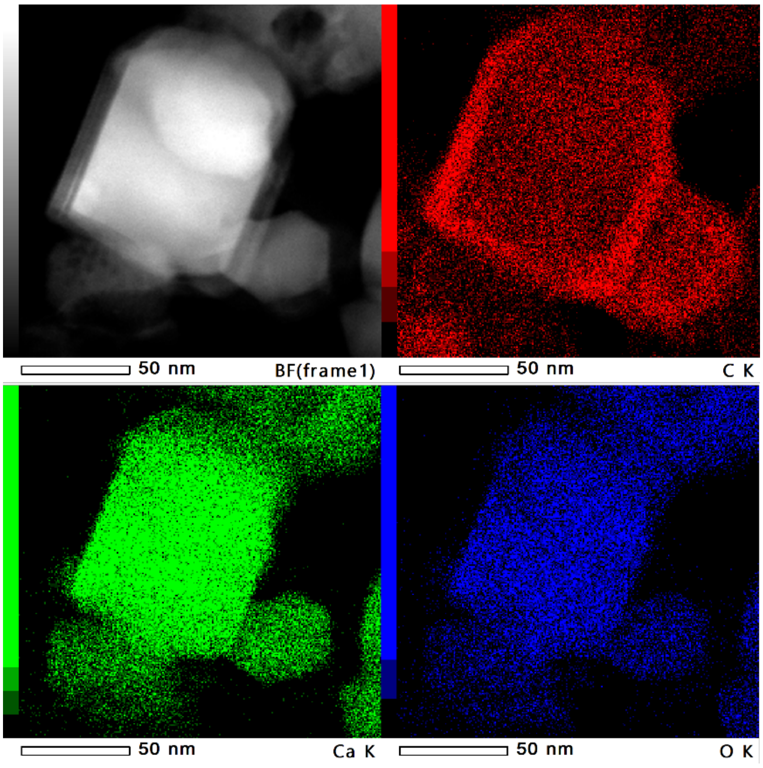

Figure S2. EDX analysis of the calcium-impregnated cellulose biochar carbonized at 1400 °C.

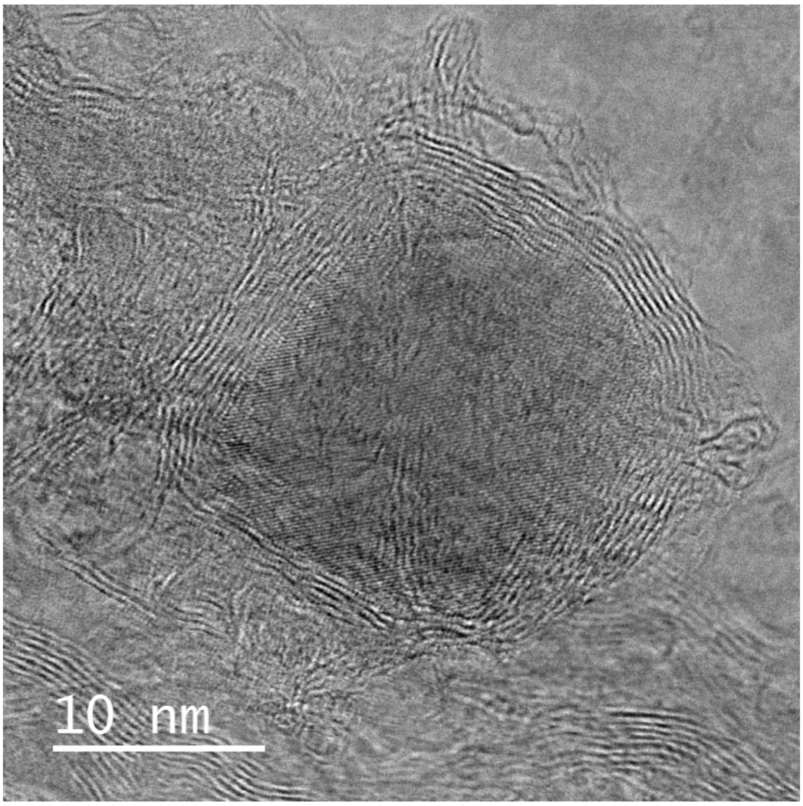

Figure S3. TEM image of the calcium-impregnated cellulose biochar at 1400 °C.

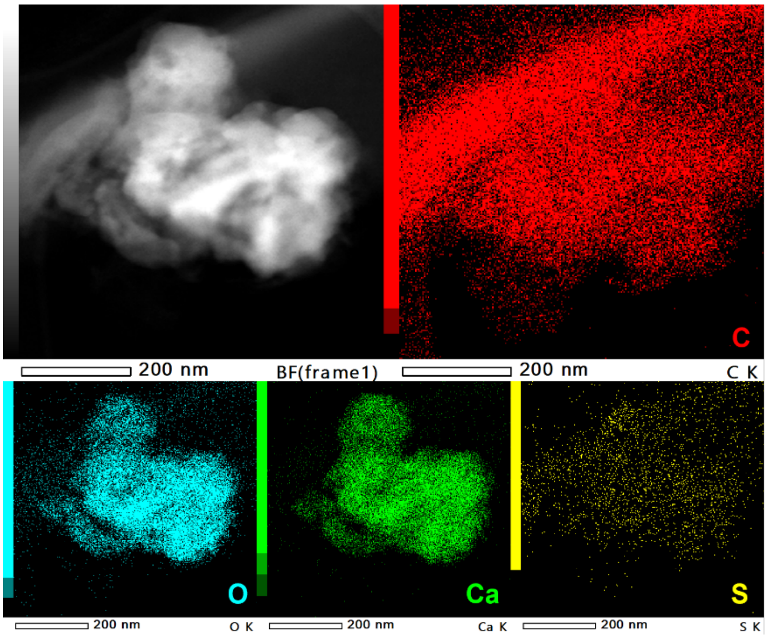

Figure S4. EDX analysis of the calcium-impregnated lignin biochar carbonized at 1000 °C.

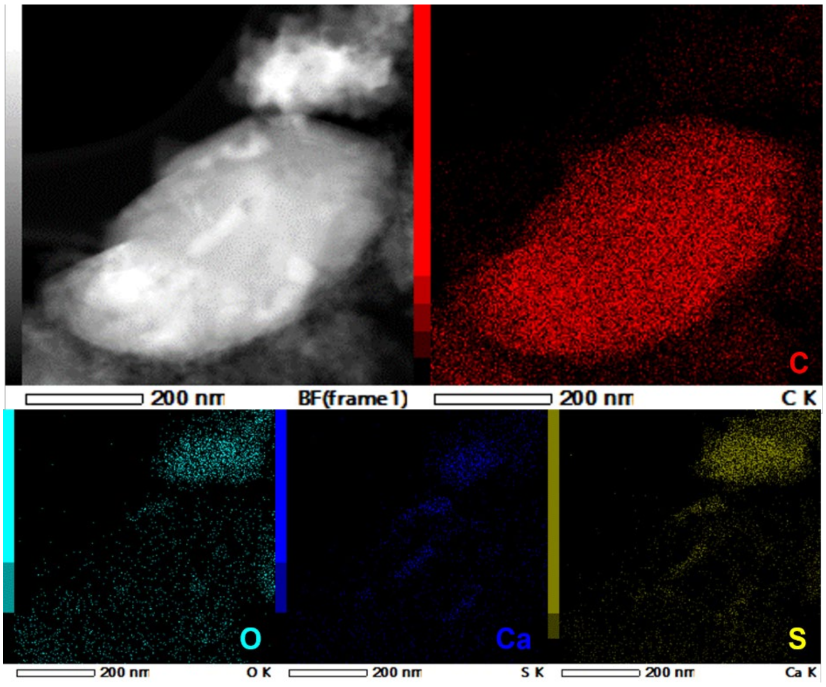

Figure S5. EDX analysis of the calcium-impregnated lignin biochar carbonized at 1400 °C.
